# Inhibition of 13-cis retinoic acid-induced gene expression of reactive-resistance genes by thalidomide in glioblastoma tumours *in vivo*

**DOI:** 10.18632/oncotarget.4727

**Published:** 2015-07-30

**Authors:** Dusan Milanovic, Carsten Sticht, Manuel Röhrich, Patrick Maier, Anca-L. Grosu, Carsten Herskind

**Affiliations:** ^1^ Department of Radiation Oncology, University Hospital Freiburg, Freiburg, Germany; ^2^ German Cancer Consortium (DKTK), Freiburg, Germany; ^3^ German Cancer Research Center (DKFZ), Heidelberg, Germany; ^4^ Centre for Medical Research, Universitaetsmedizin Mannheim, Medical Faculty Mannheim, Heidelberg University, Mannheim, Germany; ^5^ Department of Neuropathology, Institute of Pathology, Ruprecht-Karls-University Heidelberg, Heidelberg, Germany; ^6^ Department of Radiation Oncology, Universitaetsmedizin Mannheim, Medical Faculty Mannheim, Heidelberg University, Mannheim, Germany

**Keywords:** glioblastoma, 13-cis retinoic acid, thalidomide, hypoxia, angiogenesis

## Abstract

The cell differentiation potential of 13-cis retinoic acid (RA) has not succeeded in the clinical treatment of glioblastoma (GBM) so far. However, RA may also induce the expression of resistance genes such as HOXB7 which can be suppressed by Thalidomide (THAL). Therefore, we tested if combined treatment with RA+THAL may inhibit growth of glioblastoma *in vivo*. Treatment with RA+THAL but not RA or THAL alone significantly inhibited tumour growth. The synergistic effect of RA and THAL was corroborated by the effect on proliferation of glioblastoma cell lines *in vitro*. HOXB7 was not upregulated but microarray analysis validated by real-time PCR identified four potential resistance genes (IL-8, HILDPA, IGFBPA, and ANGPTL4) whose upregulation by RA was suppressed by THAL. Furthermore, genes coding for small nucleolar RNAs (snoRNA) were identified as a target for RA for the first time, and their upregulation was maintained after combined treatment. Pathway analysis showed upregulation of the Ribosome pathway and downregulation of pathways associated with proliferation and inflammation. In conclusion, combined treatment with RA + THAL delayed growth of GBM xenografts and suppressed putative resistance genes associated with hypoxia and angiogenesis. This encourages further pre-clinical and clinical studies of this drug combination in GBM.

## INTRODUCTION

Despite considerable advances in understanding the molecular biology of glioblastoma multiforme (GBM), and in developing new targeted therapies [1], the prognosis of patients with this aggressive, incurable disease remains poor. Since invariably tumours recur resulting in a median survival time of 14.6 months even with the best current treatment [2] new therapeutic approaches are urgently needed.

13-cis Retinoic Acid (RA) is a metabolite of Vitamin A which plays a fundamental role in normal embryological development [3]. Owing to its properties as an inducer of differentiation [4, 5] it has been assumed that treatment with RA may lead to less malignant behaviour and thus improved clinical outcome in GBM. However, in spite of promising experimental evidence on its activity *in vitro* [6] treatment of GBM patients with RA mostly failed to show the expected benefit in clinical settings [7, 8]. The causes for the lack of a therapeutic effect of RA are not well understood, but it has been presumed that the effectiveness of RA is limited due to its fast metabolism, decreased expression of retinoic acid receptor-β (RAR-β) [9], or methylation and thus silencing of the RAR-β promoter [10]. On the other hand, treatment of the human GBM cell line U343 with RA induced expression of different genes (HOXB7, FGF2, VEGF, and IL-8) that may contribute to tumour cell proliferation, hypoxia, and angiogenesis, thereby potentially limiting the therapeutic efficacy of RA [11]. However, induction of HOXB7 and FGF2 was suppressed when RA-treatment was combined with thalidomide (THAL) [11, 12]. THAL is an immunomodulatory drug which has been approved for treatment of multiple myeloma. Based on its anti-angiogenic effects it has been hypothesized, that THAL might also show antineoplastic effects in clinical settings [13]. However, treatment with THAL mostly failed to show any influence on the course of disease in patients with various solid tumours including GBM [14].

Based on the fact that expression of several genes which are induced by RA may be inhibited by THAL we propose that combination treatment with THAL and RA may suppress the undesirable effects of RA monotherapy and lead to improved tumor response. Thus, the purpose of the present study was to determine the effect of monotherapies with RA or THAL as well as the combination therapy in mice bearing U251 human GBM xenografts. To study possible mechanisms involved in the interaction of both substances on transcriptional control, expression of various genes in the tumour tissues was studied. We found that neither of the two monotherapies influenced growth of U251 human GBM xenografts whereas combined treatment with the two agents significantly delayed tumour growth. Gene expression analysis showed no effect of these compounds on HOXB7 in tumours excised after the end of the treatment. However, among the genes upregulated by RA, THAL suppressed the upregulation of IL-8, IGFBP-3, HILPDA, and ANGPTL4 which are associated with hypoxia and angiogenesis. Furthermore, we observed that treatment with RA as a sole compound or in combination with THAL caused upregulation of genes encoding small nucleolar RNAs (snoRNA), indicating that snoRNAs may be important transcriptional targets of RA in GBM.

## RESULTS

**Figure 1:**
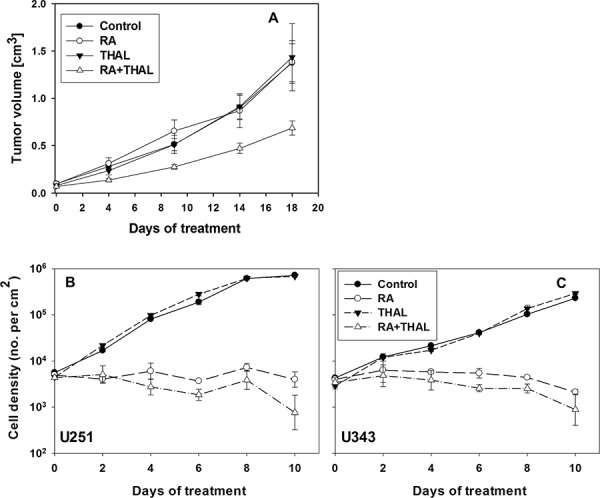
The effect of RA and THAL as sole agents or in combination on U251 xenografts **A.** Volume growth as function of time during treatment. The treatment of animals with RA or THAL as a sole compound did not affect xenograft growth in comparison to untreated controls. Treatment with RA in combination with THAL caused significant growth delay of tumours in comparison to control or animals which were treated with RA or THAL as sole compounds. Mean values and standard errors are shown. The effect of RA and THAL on cell proliferation *in vitro* **B, C.** The treatment of U251 (B) or U343 (C) with RA inhibited cell proliferation in comparison to untreated cells. The treatment with THAL as a sole compound did not affect cell proliferation in comparison to untreated controls but a significant sensitizing effect of THAL in combination with RA (*P* = 0.0007 and *P* = 0.015 for U251 and U343, respectively for days 4–10) was observed in comparison to cells treated with RA only.

The treatment with RA and THAL, as sole agents or in the combination, was well tolerated. No side effects were observed. The weight of all animals remained constant for the whole experimental period (data not shown). Treatment with RA or THAL as sole agents, did not affect the tumour growth in comparison to untreated controls. By contrast, combined treatment of RA and THAL significantly reduced tumour growth compared to untreated controls (*p* = 0.0043), and treatment with RA or THAL alone (Figure 1A).

To validate the synergistic effect of RA and THAL the effect on cell proliferation *in vitro* was tested for the U251 and U343 cell lines by scoring cell density under the microscope. RA was found to inhibit proliferation in U251 and U343 cells while THAL showed no effect. THAL in combination with RA showed a sensitizing effect resulting in a reduction of the cell density relative to RA alone (Figure 1B, 1C). Of note, the cell size was observed to be increased after 8–10 days of treatment with RA, and in particular with RA+THAL (not shown).

Since RA was previously shown to upregulate HOXB7 expression, and upregulation could be suppressed by THAL, we tested the expression of this gene in tumours excised at the end of the 18-day treatment period. No upregulation was detected in tumours treated with RA, although a non-significant trend (*P* = 0.13) for a minor downregulation was detected for tumours treated with THAL alone or in combination with RA (Supplementary Figure). However, this result does not exclude a transient effect earlier during treatment that might have disappeared by the time when RNA was isolated. In order to explore alternative genes associated with the effect of combined RA + THAL treatment on tumour growth, microarray analysis was performed.

**Figure 2:**
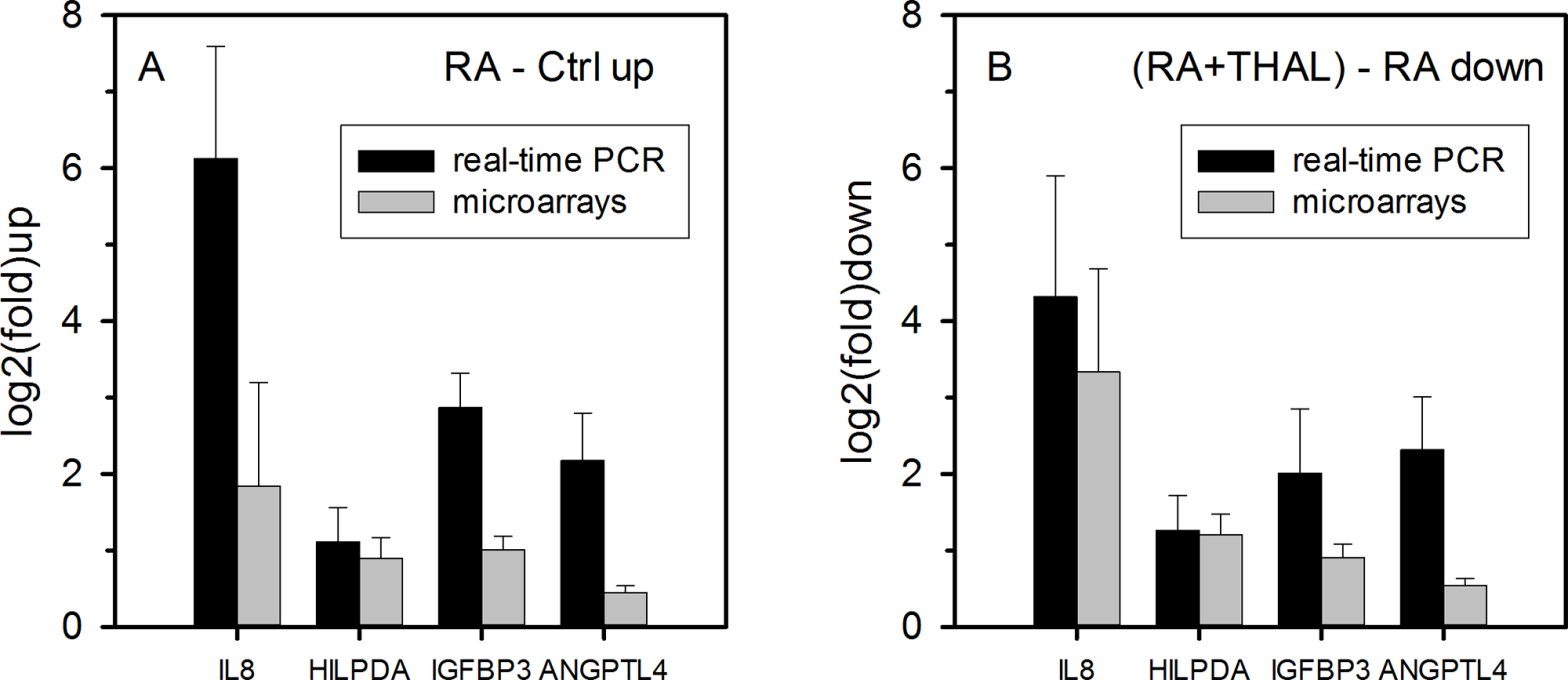
Gene expression analysis of RNA isolated from tumours after treatment **A.** Validation by real-time PCR of the expression of RA-induced candidate genes selected from microarray analysis on the basis that their upregulation in tumours treated by RA was suppressed in tumours treated with RA + THAL. log2(fold) upregulation relative to control tumours (mean values and ranges for 2 tumours) are shown. Upregulation measured by real-time PCR was at least as strong determined from microarrays. **B.** log2(fold) downregulation of gene expression in tumours treated with RA + THAL relative to tumours treated with RA alone (mean values and ranges). Downregulation by RA + THAL relative to RA was at least as strong as determined by microarray analysis and comparable with upregulation by RA alone, confirming efficient suppression by adding THAL to RA.

Owing to the small number of tumours available for analysis, significance levels were generally modest. Nevertheless, based on the hypothesis that RA induces expression of genes that compromise the anti-tumour effect of RA, genes suppressing RA-induced upregulation were filtered. IL-8 showed the strongest (10-fold) mean suppression (*P* = 0.037, *n* = 3) by the combined treatment RA+THAL relative to induction by RA alone. Among the top-20 genes with the strongest suppression of RA-upregulated genes by THAL, the three genes with the highest significance scores were HILPDA (2.3-fold, *P* = 0.0017, *n* = 3), IGFBP3 (1.9-fold, *P* = 0.0006, *n* = 3), and ANGPTL4 (1.5-fold, *P* = 0.0002, *n* = 3). In order to validate the differential expression, real-time PCR was performed. Although high-quality RNA was available only for two tumours per treatment (three for the controls) at the time of the assay, upregulation by RA was validated for all four genes and THAL downregulated the genes by approximately the same fold reduction. This supports the hypothesis that THAL suppresses RA-induced expression of these genes (Figure 2A, 2B).

**Figure 3:**
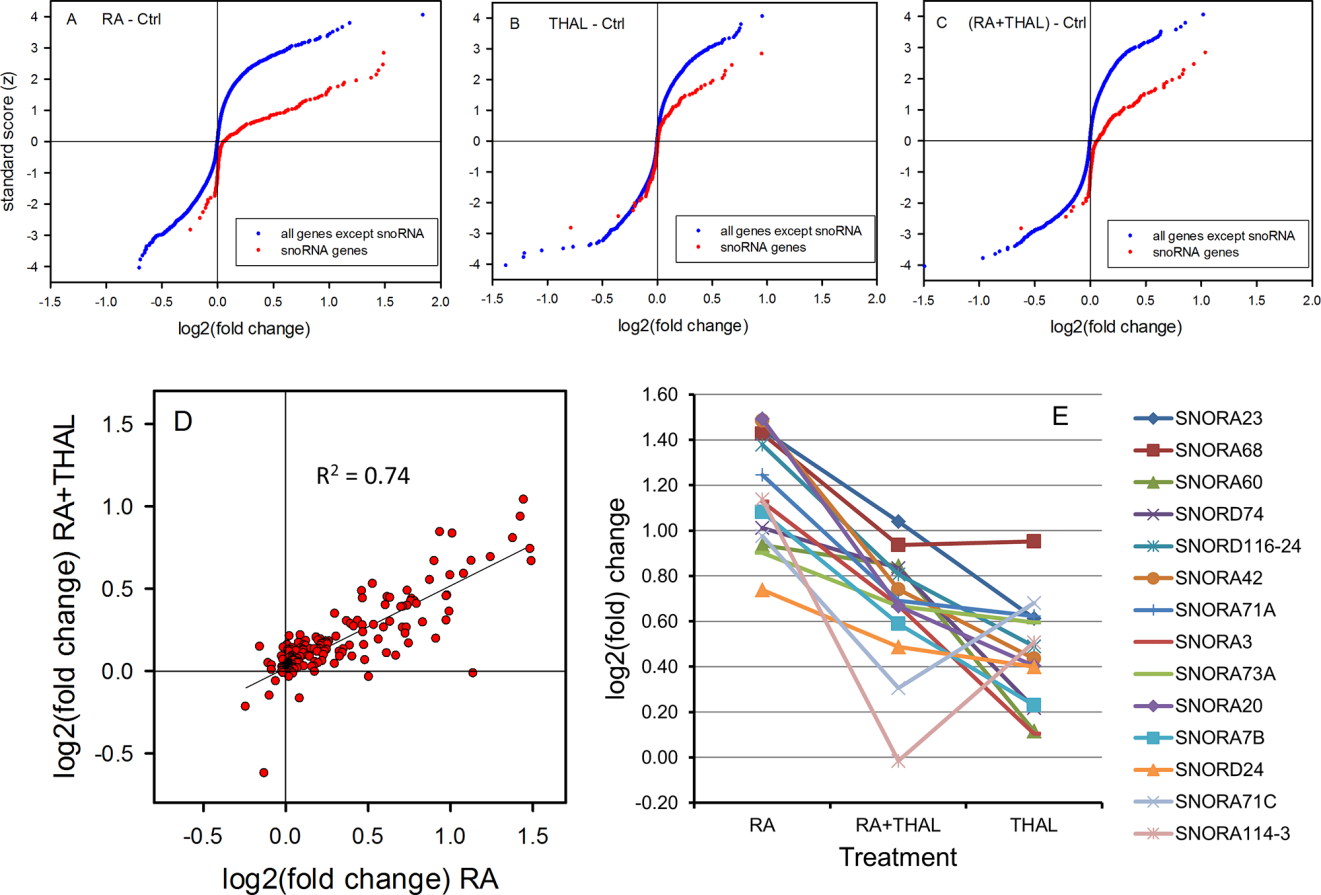
Differential expression of snoRNA genes compared with all other genes in tumours after treatment with RA, THAL or the combination RA + THAL Cumulative distribution of down-and upregulation shown as the Normalized Score versus log2(fold) change in tumours treated with **A.** RA, **B.** THAL, and **C.** RA + THAL relative to untreated controls. RA caused upregulation of approximately 50%, and THAL of approximately 25% of the snoRNA genes. RA + THAL upregulated aproximately the same fraction of snoRNA genes as RA although the degree of upregulation was reduced. **D.** Upregulation of snoRNA by RA + THAL correlated well with upregulation by RA alone. **E.** Upregulation by RA-THAL was intermediate between the levels in RA and THAL tumours for the majoríty of the most upregulated snoRNA genes.

While THAL may prevent upregulation of certain RA target genes, we looked for other genes that might be differentially expressed after combined RA + THAL treatment relative to single treatment with RA or THAL. Tumours treated with RA alone showed >1.5-fold upregulation of 80 genes and >1.5-fold downregulation of 12 genes compared with untreated control tumours. Interestingly, genes coding for small nucleolar RNA (snoRNA) were greatly overrepresented among up- but not downregulated genes (Figure 3A). Thus approximately half of the 214 snoRNAs on the microarray chip were upregulated while very few were downregulated. Tumours treated with THAL alone showed >1.5-fold upregulation of 20 genes and >1.5-fold downregulation of 9 genes. In this case, overrepresentation of snoRNA genes involved approximately a quarter of the snoRNA genes for upregulated genes while downregulated snoRNA genes were observed at the same frequency as other genes (Figure 3B).

In tumours receiving the combined treatment RA + THAL, 21 genes were upregulated >1.5 fold and 19 genes were downregulated >1.5 fold. Further analysis of the microarrays showed upregulation of approximately half of the snoRNA genes (Figure 3C). Although the fold upregulation was smaller than after RA alone, there was an excellent correlation (R^2^ = 0.74) between upregulation by combined RA + THAL treatment and RA alone (Figure 3D). The correlations was much weaker between snoRNA levels in THAL and RA + THAL-treated tumours (R^2^ = 0.32) although a moderate correlation between RA and THAL (R^2^ = 0.43) was observed. Notably, the top-10 upregulated snoRNA in RA + THAL treated tumours showed a high degree of overlap with the top-10 upregulated snoRNA after single treatment with RA or THAL (8 out of 10 genes in both cases; Table 1). Comparison of the expression levels showed intermediate levels in RA+THAL treated tumours relative to the higher levels in RA and the lower levels in THAL-treated tumours for most of the 14 snoRNA genes (Figure 3E). A linear model analysis of the relative effects of RA and THAL on combined treatment with RA+THAL for all 214 snoRNA genes showed a highly significant influence of RA-induced snoRA expression (*P* < 0.0001), and no influence of THAL-induced snoRA expression (*P* = 0.94; power = 0.98 for an influence <5% that of RA).

**Table 1:** log2(fold change) and rank of snoRNA genes among the top-10 upregulated SNORs in tumours after at least one of the treatments, RA and THAL as sole agents, or in the combination RA + THAL

	log2(fold change)			Rank		
Gene	RA-Ctrl	(RA + THAL)-Ctrl	THAL-Ctrl	RA	RA + THAL	THAL
SNORA23	1.45	1.04	0.61	3	1	4
SNORA68	1.43	0.94	0.95	4	2	1
SNORA60	0.94	0.84	0.12	16	3	41
SNORD74	1.01	0.83	0.22	10	4	19
SNORD116–24	1.38	0.81	0.49	5	5	7
SNORA42	1.48	0.74	0.44	2	6	8
SNORA71A	1.24	0.69	0.62	6	7	3
SNORA3	1.13	0.67	0.10	8	8	43
SNORA73A	0.90	0.67	0.60	18	9	5
SNORA20	1.49	0.67	0.40	1	10	9
SNORA7B	1.08	0.59	0.23	9	11	18
SNORA24	0.74	0.49	0.40	26	15	10
SNORA71C	0.98	0.31	0.68	15	32	2
SNORD114–3	1.14	-0.01	0.51	7	206	6

A considerable degree of overlap was observed: 6 of 14 genes were in the top-10 after all three treatments.

**Figure 4:**
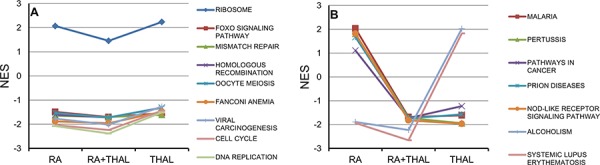
Pathway analysis (GSEA) for genes regulated by RA, THAL, or RA+THAL The Normalized Enrichment Score (NES) is compared for treatment by RA, RA + THAL, and THAL. **A.** Pathways showing similar up- or downregulation in tumours treated by RA and THAL alone. NES tended to be slightly lower in RA + THAL treated tumours. **B.** Pathways showing opposite differential regulation in tumours treated RA and THAL alone (up vs. down, or down vs. up). Five pathways (Malaria, Pertussis, Pathways in Cancer, Prion Diseases, Nod-like Receptor Signaling Pathway) showed the same down-regulation in RA + THAL as in THAL-treated tumours, while two pathways (Alcoholism, Systemic Lupus Erythematosus) showed the same down-regulation in RA + THAL as in RA-treated tumours.

Gene Set Enrichment Analysis (GSEA) showed no upregulated pathways with FDR <0.25 for RA + THAL whereas several pathways were significantly downregulated. A list of pathways with FDR <0.05 is shown in Supplementary Table 1. DNA Replication, and Cell Cycle, were among the most downregulated pathways in RA + THAL as well as RA-treated tumours. Similarly, Systemic Lupus Erythematosis (SLE), and Alcoholism were strongly downregulated with FDR <0.003 after both treatments. The leading edge genes in the latter two pathways was dominated by histone genes (75–95%) and may thus reflect an effect on proliferation. Interestingly, several DNA repair pathways showed FDR <0.05 in RA + THAL tumours. A comparison of the normalised enrichment score (NES) for selected pathways is shown for pathways downregulated by RA and THAL as sole agents (Figure 4A), and pathways upregulated by RA but downregulated by THAL, or vice versa (Figure 4B). The Ribosome pathway was enriched in all treated tumours though not significantly after combined treatment. Pathways associated with the cell cycle, DNA replication, repair, and cell stress (FOXO), were consistently enriched with negative scores after all three treatments. SLE and Alcoholism pathways were enriched after all three treatments but with positive NES in THAL and negative in RA and RA+THAL tumours. By contrast, four pathways associated with the immune response (Malaria, Pertussis, Prion Diseases, and NOD-like Receptor Signaling), as well as ‘Pathways in Cancer’, showed positive NES in RA but negative in THAL and RA + THAL treated tumours. Thus, down-regulation by RA dominated over upregulation by THAL for the two pathways related to proliferation while down-regulation by THAL dominated over upregulation by RA for the pathways associated with inflammation.

## DISCUSSION

In the present study we tested the influence of RA and THAL as mono- or combination therapies on the growth of U251 human GBM xenografts. Combined treatment caused significant delay in tumour growth whereas the no effect was observed for either agent on its own. Our hypothesis was that RA administration will induce expression of HOXB7 which should be suppressed by THAL for combined treatment with the two drugs [11]. Surprisingly we did not observe induction of HOXB7 in tumours treated with RA. However, RNA was isolated at the end of treatment and it is possible that HOXB7 expression was transiently induced at earlier time points during treatment with RA.

Based on experimental evidence that RA may inhibit proliferation of glioma cells in a dose dependent manner [17] and induces differentiation of various cell types [4, 18] it has been expected that RA may have a positive impact on the survival of GBM patients. The mechanism by which retinoids inhibit the proliferation of GBM cells is not well understood but it has been shown in different glioma cell lines that the epidermal growth factor receptor (EGFR) can be targeted by all-trans retinoic acid (ATRA). This resulted in inhibition of autophosphorylation of EGFR and not in downregulation of expression of EGFR [6]. In two human GBM cell lines, T98G and U87MG, treatment with RA induced astrocytic differentiation with upregulation of glial fibrillary acidic protein (GFAP) and downregulation of telomerase activity [19]. The results from our *in vitro* experiments confirmed that proliferation of the U251 and U343 cell lines was inhibited by RA, which was accompanied by an increase in cell size indicating differentiation of the cells. In previous experiments [11] we found a higher sensitivity of the cells to RA in clonal culture compared with mass culture conditions. Thus differences in cell density might contribute to the higher sensitivity to RA observed *in vitro* compared with tumours *in vivo*. However, in both cases a synergistic effect was observed for the combination of THAL with RA consistent with previous findings from a colony formation assay [11]. Therefore, it remains likely that upregulated expression of protective genes in response to treatment with RA as a sole agent may limit the efficacy of RA. We identified IL8, IGFBP-3, ANGPTL4, and HILDPA, as RA-upregulated genes that were suppressed by simultaneous treatment with THAL. These candidate protective genes are associated with hypoxia and angiogenesis which are known to cause resistance to tumour therapy [20, 21]. As proposed in the action-reaction model [22] drug treatment of tumour cells may cause not only the desired effect (action) but may also induce negative effects (reaction). In the present case, treatment with RA may lead to differentiation of tumor cells rendering them less aggressive but induction of resistance genes such IL-8, HILDPA, IGFBPA, and ANGPTL4 may have a negative impact on the neoplastic process causing stimulation of angiogenesis and further progression. Based on the present study, the addition of THAL in combination with RA may abrogate the “reaction” caused by RA and lead to better tumour response.

Multiple hypoxic areas, which normally can be found in GBM, play a crucial role in the development of therapy resistance and the activation of angiogenesis. IL-8 is a member of the chemokine family which plays and important role in gliomagenesis and angiogenesis [23] and it has been found to be induced by RA in GBM and neuroblastoma cell lines [11, 24]. It is highly expressed in pseudopalisading GBM cells surrounding necrotic areas [25] indicating that hypoxia/anoxia may be an important stimulus for its expression [23].

IGFBP-3 is a part of the insulin-like growth factor (IGF) signaling pathway [26]. In patients with newly diagnosed GBM, elevated expression of IGFBP-3 was associated with shorter survival [27]. In U373 GBM cells, IGFBP-3 was identified as secretory protein under hypoxic conditions [28]. In U251 cells *in vitro*, IGFBP-3 knockdown significantly diminished proliferation, motility, migration and invasive capacity [29]. These authors also showed that exogenous overexpression of IGFBP-3 regulates STAT-1 expression, one of the strong predictor of poor survival in patients with GBM. Recently, it was shown that the insulin-like growth factor-1 receptor (IGF-1R), is the major receptor tyrosine kinase (RTK) driving mitogenic signaling in U251 cells *in vitro* [30]. Interestingly, IGF-1R signaling is regulated by IGFBP-3 [31].

In neoplastic disease, Angiopoietin-like 4 (ANGPTL4) acts as a negative regulator of apoptosis, regulates angiogenesis and promotes tumour invasion and metastasis [32]. Hypoxia can induce ANGPTL4 by up-regulation of the transcription factor hypoxia-inducible factor-1α (HIF-1α) [33]. In GBM LN229-vIII tumour xenografts, EGFRvIII induces ANGPTL4 expression through the ERK/c-Myc pathway and promotes tumour angiogenesis [34]. These findings suggest that ANGPTL4 may be a fundamental modulator of angiogenesis in a hypoxic tumour microenvironment.

Hypoxia inducible lipid droplet-associated (HILPDA; formerly known as Hypoxia-inducible protein 2, HIG2) is also induced by hypoxia [35]. In a mouse colorectal cancer orthotopic model, over-expression of HIG2 promoted tumour growth by suppressing apoptosis [36]. Renal cell carcinoma patients show increased plasma concentration of HILPDA comparable with healthy volunteers on patients with chronic glomerulonephritis [37]. To the best of our knowledge, the present study is the first reporting the expression of HILPDA in GBM.

Upregulation of a large number of snoRNA was observed in tumours treated with RA alone. To our knowledge, this is the first report on snoRNA as important transcriptional targets of RA. Although expression levels were reduced by approximately half in tumours treated with the combination of RA + THAL, the expression was predicted entirely by the expression induced by RA alone. Among the ten most upregulated genes, five (SNORA20, SNORA71A, SNORA23, SNORA3, SNORA68) were recently found to be downregulated by HOXA10 in LN18 glioblastoma cells [38]. Since HOXA10 expression is associated with therapy resistance of glioblastoma [39], upregulation of snoRNA by RA, alone or in combination with THAL, might contribute to decreasing therapy resistance to chemo- or radiotherapy. Pathway analysis suggested that the Ribosome pathway was upregulated by RA or THAL as sole treatments although the effect was reduced after combined treatment. On the other hand, it has been reported that in multiple myeloma cells internal ribosome entry site (IRES) of bFGF was inhibited at low concentration of THAL [40].

The upregulation of many snoRNAs may be related to the upregulation of the Ribosome pathway since processing of rRNA is a major function of snoRNAs [41]. In this respect, RA and THAL showed a degree of antagonism to each other because combined treatment resulted in less upregulation of snoRNAs and the Ribosome pathway than RA alone, even though both gene sets were also upregulated by THAL on its own. Downregulation by RA + THAL was pronounced for pathways related to proliferation and DNA repair and could be ascribed to RA, consistent with its differentiation-inducing activity. Furthermore, THAL was responsible for downregulating pathways related to inflammation, consistent with its known antiinflammatory function. We speculate that the upregulation of snoRNAs and the ribosome pathway may be associated with induction of differentiation and that RA and THAL may affect different subsets of differentiation-related genes.

**Figure 5:**
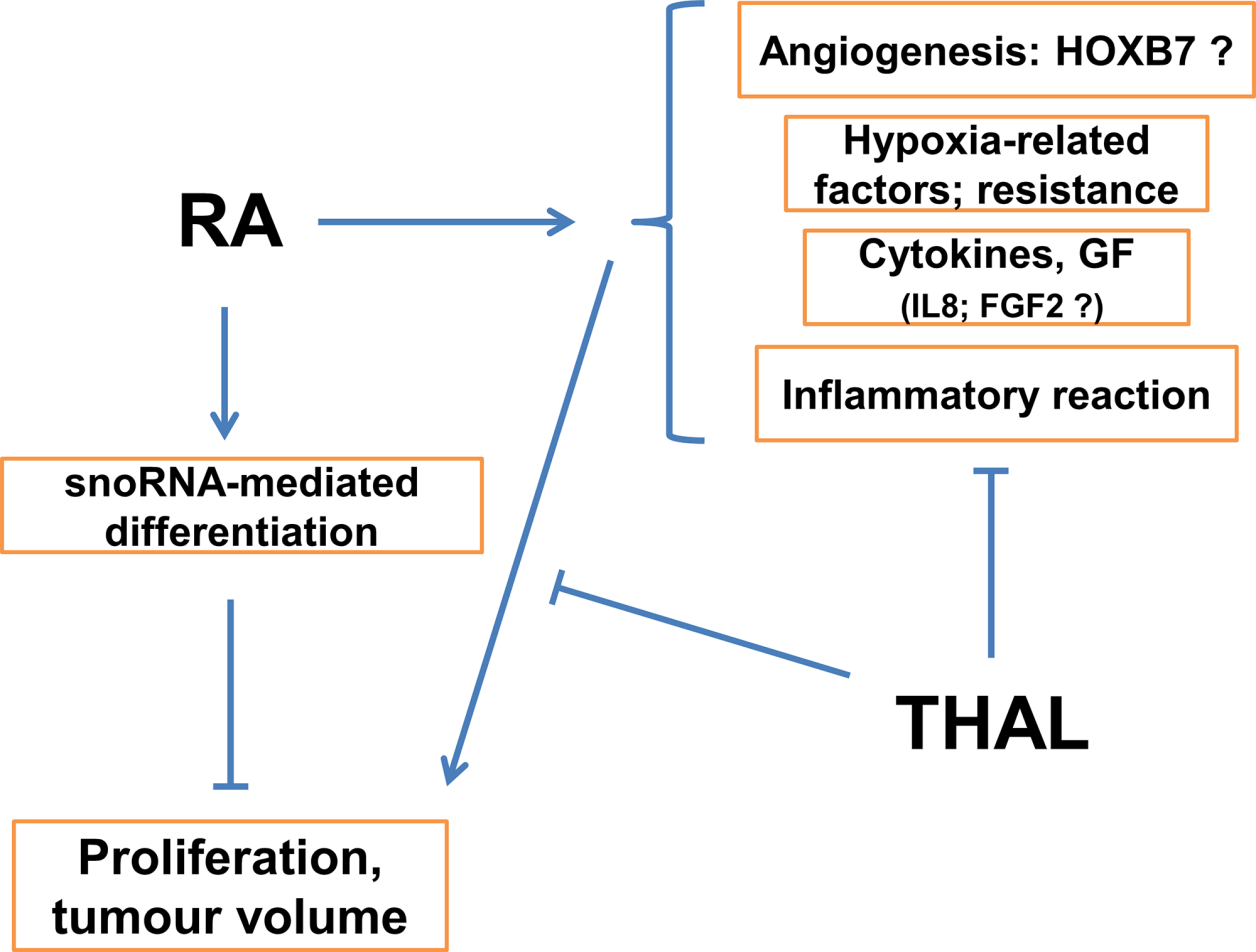
Schematic model of the hypothetical mechanism for tumour growth inhibition by combined treatment with RA + THAL

In addition to the interactions between RA and THAL, it cannot be excluded that an increase in the volume of cells and extracellular matrix by RA-induced differentiation may contribute to the lack of an effect on tumour volume by treatment with RA alone. Figure 5 shows a schematic diagram of the possible mechanisms involved in the positive effect of combined treatment with RA and THAL on tumour growth.

Despite the lack of relevant Phase III clinical trials, it can be concluded that treatment with RA or THAL as sole compounds or in combination with other chemotherapeutics or radiotherapy, showed limited efficacy in GBM patients. Possible reasons for inefficacy of RA are discussed above. It seems likely that efficacy of THAL requires upregulation of certain target genes as induced by RA. In a recent phase II multifactorial clinical study [42], GBM patients who had completed standard radiochemotherapy with temozolomide (TMZ) were treated in an adjuvant setting with dose-dense (dd) TMZ combined with different, non-classical antineoplastic drugs. Generally all treatments were well tolerated, although high rates of lymphopenia were observed, but patients who received RA as partly of the combination with ddTMZ showed significantly shorter median survival compared to other groups. In order to retain the positive effect of standard radiochemotherapy with concurrent and conventional adjuvant TMZ on survival, we suggest that the growth inhibitory effect of combined RA+THAL treatment should be explored in the adjuvant setting by alternating RA + THAL with standard TMZ in the weeks where adjuvant TMZ is not administered.

In summary, we have demonstrated for the first time that combined treatment with RA and THAL delayed growth of human U251 GBM xenografts. Mechanistically, we observed that genes associated with hypoxia and angiogenesis were induced by RA treatment, and that this induction was successfully suppressed by adding THAL to the treatment. We suggest that the combination of RA and THAL may have a positive impact on the efficacy of RA and survival of these patients by inhibiting RA-induced resistance genes. To explore this hypothesis further and to optimize treatment schedules, further pre-clinical and clinical studies should be performed.

## MATERIALS AND METHODS

### Animals

All animal experiments were performed in accordance with the German Animal License Regulations and were approved by the animal care committee of the regional authority in Freiburg (registration number: G-08-59). Athymic female nude mice NMRI-Foxn1^nu^ (aged 6 weeks) were obtained from Taconic Biosciences Inc. (Ry, Denmark) and tumours were induced by inoculation of 1.5 × 10^6^ human glioblastoma (U251) cells s. c. into the right hind limb. Palpable U251 xenografts developed within 8 to 10 days. The U251 cell line was obtained from the tumor bank of the National Cancer Institute (NCI), Frederick, Maryland. Cells were grown as a monolayer in RPMI-1640 culture medium supplemented with 10% foetal bovine serum (FBS; Biochrom, Berlin, Germany) at 37°C under 5% CO_2_.

### Drug treatment and tumour growth delay

13-cis retinoic acid (RA) and thalidomide (THAL) (Sigma Aldrich, Taufkirchen, Germany) were dissolved in refined sesame oil (Henry Lamotte Oils GmbH, Bremen, Germany) immediately before administration. Animals were randomly assigned in 4 groups with 7 to 11 animals per group: control, RA, THAL, and RA + THAL and treated daily (Monday-Friday) via intragastric tube with RA (30 mg/kg), THAL (30 mg/kg) or their combination for 18 days. Animal weight was measured daily. The tumour volume was determined by external caliper measurement of the greatest longitudinal diameter (length) and the greatest transverse diameter (width). Tumour volume based on caliper measurements was calculated by the modified ellipsoidal formula: Tumour volume = 0.5 × (length × width)^2^.

Animals were sacrificed following the IACUC guidelines one day after the last treatment and tumour xenografts were resected and subsequently frozen or fixed with formalin.

### Cell proliferation *in vitro*

The U251 and U343 cell lines (obtained from ATCC, LGC Promochem, Wesel, Germany, and the tumour bank of the German Cancer Research Centre, Heidelberg, Germany, respectively) were seeded in 12-well plates (Falcon, Corning, Wiesbaden, Germany) at 1 × 10^4^ cells per well and incubated in RPMI-1640 medium supplemented with 10% fetal bovine serum (FBS; both from Biochrom, Berlin, Germany) under 5% CO_2_ at 37°C. After 24 h, the cultures were treated with 30 μg/ml RA, 30 μg/ml THAL, or a combination, dissolved in DMSO (0.2%; final concentrations) with DMSO added to control cultures. Drugs and medium were replenished every second day and plates were fixed after 0, 2, 4, 6, 8, and 10 days of treatment. Cell nuclei were stained with DAPI, and the number of cells were scored in ten 0.5 × 0.7 mm frames along the diameter of the well using a fluorescence microscope with 200x magnification. Bright-field images were used for verification. Three independent experiments were performed.

### Gene expression analysis

Total RNA was extracted and purified from <50 mg slices of snap-frozen tumours using the RNeasy mini kit (Qiagen, Hilden, Germany) according to the manufacturer's instructions. First strand cDNA synthesis of 1 μg total RNA was performed using AMV first strand cDNA synthesis kit for RT-PCR (Roche, Mannheim, Germany) and oligo p(dT)_15_ primer according to the manufacturer's protocol. For Affymetrix whole transcript expression analysis, a total of 1 μg of RNA was transcribed to cDNA according to the protocol provided with the High Capacity cDNA Reverse Transcription kit (Life Technologies GmbH, Darmstadt, Germany). Gene expression profiling was performed using arrays of hugene-1_0-type from Affymetrix (Affymetrix Inc., Santa Clara, CA, USA). Biotinylated sense-strand DNA was prepared according to the Affymetrix standard labelling protocol and then hybridized for 16 h. Arrays were washed and stained using the Fluidics Station 450. Scanning was performed by Scanner 3000 (Affymetrix High Wycombe, UK).

### Statistical analysis

The growth of treated tumours relative to untreated control tumours was tested by the *t*-test. The significance of mitotic index and real-time PCR was tested by the non-parametric Wilcoxon/Kruskal-Wallis test. The relative influences of RA and THAL on differential snoRNA expression by RA+THAL was analysed by a linear model. The JMP 11 Statistical Discovery software package (SAS Institute GmbH, Böblingen, Germany) was used.

A Custom CDF Version 18 with entrez based gene definitions was used to annotate the arrays [15]. The raw fluorescence intensity values were normalized applying quantile normalization and RMA background correction. ANOVA was performed to identify differential expressed genes using a commercial software package SAS JMP10 Genomics, version 6, from SAS (SAS Institute, Cary, NC, USA). A false positive rate of *a* = 0.05 with False Discovery Rate (FDR) correction was taken as the level of significance.

Gene Set Enrichment Analysis (GSEA), was used to determine whether defined lists (or sets) of genes exhibit a statistically significant bias in their distribution within a ranked gene list (see http://www.broadinstitute.org/gsea/ for details [16]). Pathways belonging to various cell functions such as cell cycle or apoptosis were obtained from public external databases (KEGG, http://www.genome.jp/kegg/) The raw and normalized data are deposited in the Gene Expression Omnibus database Series accession no. GSE71224 (http://www.ncbi.nlm.nih.gov/geo/query/acc.cgi?acc=GSE71224).

## SUPPLEMENTARY FIGURE AND TABLE




