# Supplementary material for: Inhibition of 13-cis retinoic acid-induced gene expression of reactive-resistance genes by thalidomide in glioblastoma tumours in vivo
Source: Oncotarget. 2015 Jul 30;6(30):28938–48. doi: 10.18632/oncotarget.4727 (PMC4745702; doi:10.18632/oncotarget.4727)
Supplement: Supplementary file 1 [file oncotarget-06-28938-s001.pdf]

SUPPLEMENTARY FIGURE AND TABLE

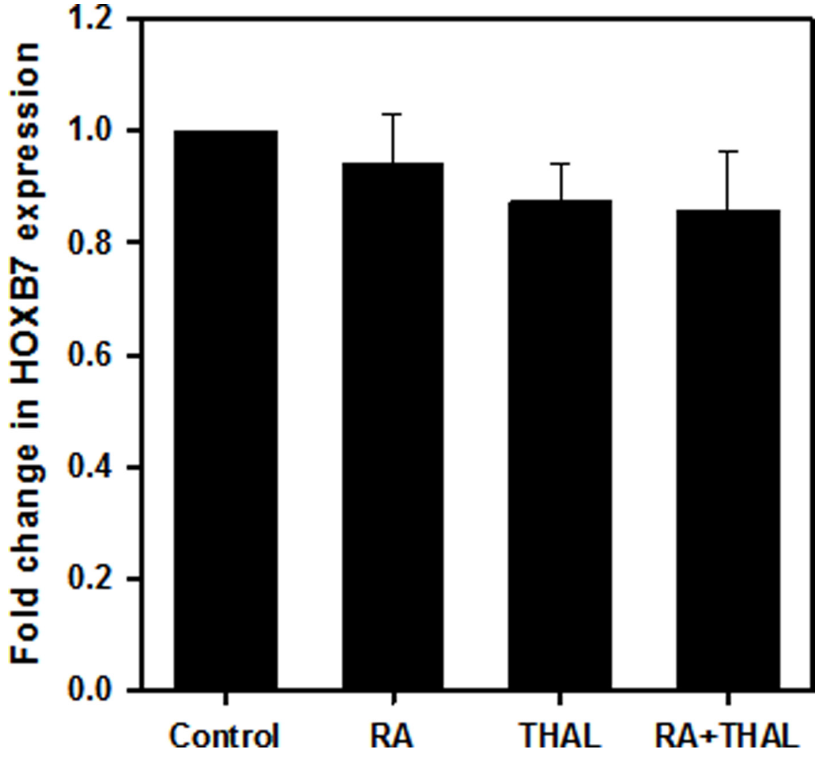

Supplementary Figure S1: Fold change in HOXB7 gene expression in tumours after treatment with RA, THAL, or the combination, RA + THAL. No significant change in expression was observed (mean values and std. errors; $n = 3$).

Supplementary Table S1: Gene Set Enrichment analysis of pathways differentially regulated in U251 xenografts treated with RA and THAL as sole agents, or in combination, RA+THAL, relattive to untreated controls. SIZE: number of genes in the pathway; ES: enrichment score; NES: normalized enrichment score; NOM *p*-val: nominal *p*-value; FDR *p*-val: false discovery rate *p*-value.
