# Supplementary material for: Inhibition of 13-cis retinoic acid-induced gene expression of reactive-resistance genes by thalidomide in glioblastoma tumours in vivo
Source: Oncotarget. 2015 Jul 30;6(30):28938–48. doi: 10.18632/oncotarget.4727 (PMC4745702; doi:10.18632/oncotarget.4727)
Supplement: Supplementary file 2 [file oncotarget-06-28938-s002.docx]

| NAME | SIZE | ES | NES | NOM p-val | FDR q-val |
| --- | --- | --- | --- | --- | --- |
| RA - up |  |  |  |  |  |
| 03010 - RIBOSOME | 118 | 0.63473403 | 2.0749853 | 0 | 0 |
| 04640 - HEMATOPOIETIC CELL LINEAGE | 83 | 0.6527768 | 2.029973 | 0 | 3.82E-04 |
| 05144 - MALARIA | 46 | 0.7156995 | 2.029006 | 0 | 2.55E-04 |
| 05134 - LEGIONELLOSIS | 54 | 0.69369376 | 2.0209043 | 0 | 1.91E-04 |
| 05150 - STAPHYLOCOCCUS AUREUS INFECTION | 48 | 0.68772525 | 1.955503 | 0 | 6.08E-04 |
| 04610 - COMPLEMENT AND COAGULATION CASCADES | 67 | 0.6342024 | 1.899561 | 0 | 0.00291701 |
| 05332 - GRAFT-VERSUS-HOST DISEASE | 36 | 0.72312486 | 1.8979158 | 0 | 0.00250029 |
| 05323 - RHEUMATOID ARTHRITIS | 85 | 0.6041314 | 1.8945374 | 0 | 0.00240006 |
| 05143 - AFRICAN TRYPANOSOMIASIS | 32 | 0.6917194 | 1.8201795 | 0 | 0.00854042 |
| 04668 - TNF SIGNALING PATHWAY | 110 | 0.5633314 | 1.8198959 | 0 | 0.00768638 |
| 05133 - PERTUSSIS | 72 | 0.6100675 | 1.8188664 | 0 | 0.00698762 |
| 04621 - NOD-LIKE RECEPTOR SIGNALING PATHWAY | 57 | 0.62665325 | 1.8180479 | 0.00190476 | 0.00654677 |
| 04672 - INTESTINAL IMMUNE NETWORK FOR IGA PRODUCTION | 45 | 0.64131105 | 1.7978747 | 0 | 0.00822498 |
| 04064 - NF-KAPPA B SIGNALING PATHWAY | 88 | 0.5591207 | 1.7525957 | 0 | 0.01466734 |
| 05020 - PRION DISEASES | 36 | 0.62676597 | 1.6705425 | 0.00787402 | 0.03768457 |
| RA - down |  |  |  |  |  |
| 03030 - DNA REPLICATION | 35 | -0.7462497 | -2.0703406 | 0 | 0 |
| 04110 - CELL CYCLE | 123 | -0.5958691 | -2.0332615 | 0 | 0 |
| 05322 - SYSTEMIC LUPUS ERYTHEMATOSUS | 115 | -0.57685345 | -1.9346104 | 0 | 0.00120522 |
| 05034 - ALCOHOLISM | 166 | -0.5301117 | -1.8902884 | 0 | 0.00270134 |
| 03460 - FANCONI ANEMIA PATHWAY | 51 | -0.6417672 | -1.8771456 | 0 | 0.00286441 |
| 04914 - PROGESTERONE-MEDIATED OOCYTE MATURATION | 86 | -0.5447923 | -1.7765465 | 0 | 0.01454881 |
| 05203 - VIRAL CARCINOGENESIS | 197 | -0.48515818 | -1.7650677 | 0 | 0.01465192 |
| 00310 - LYSINE DEGRADATION | 48 | -0.583185 | -1.7020247 | 0 | 0.03540405 |
| 05212 - PANCREATIC CANCER | 66 | -0.54747045 | -1.6965925 | 0.0020202 | 0.03334174 |
| 03430 - MISMATCH REPAIR | 23 | -0.66019595 | -1.6502612 | 0.01171875 | 0.04929015 |

| NAME | | SIZE | ES | NES | NOM p-val | FDR q-val |
| --- | --- | --- | --- | --- | --- | --- |
| THAL - up | |  |  |  |  |  |
| 03010 - RIBOSOME | | 118 | 0.63942504 | 2.2458894 | 0 | 0 |
| 05034 - ALCOHOLISM | | 166 | 0.54919595 | 2.0221646 | 0 | 0.00133165 |
| 00190 - OXIDATIVE PHOSPHORYLATION | | 117 | 0.55429393 | 1.953723 | 0 | 0.00180891 |
| 05322 - SYSTEMIC LUPUS ERYTHEMATOSUS | | 115 | 0.5211434 | 1.8233005 | 0 | 0.01065174 |
| 05012 - PARKINSON'S DISEASE | | 127 | 0.50470155 | 1.8053097 | 0 | 0.01135675 |
| 00910 - NITROGEN METABOLISM | | 17 | 0.7439091 | 1.8051248 | 0.0019685 | 0.00946396 |
| THAL - down | |  |  |  |  |  |
| 04622 - RIG-I-LIKE RECEPTOR SIGNALING PATHWAY | | 69 | -0.62852997 | -2.005055 | 0 | 0.00391331 |
| 04621 - NOD-LIKE RECEPTOR SIGNALING PATHWAY | | 57 | -0.63995457 | -1.9675208 | 0 | 0.00321204 |
| 05133 - PERTUSSIS | | 72 | -0.60752666 | -1.9463699 | 0 | 0.0039081 |
| 05150 - STAPHYLOCOCCUS AUREUS INFECTION | | 48 | -0.6611987 | -1.9454675 | 0 | 0.00293108 |
| 05134 - LEGIONELLOSIS | | 54 | -0.63012844 | -1.9324397 | 0.00179211 | 0.00337298 |
| 05168 - HERPES SIMPLEX INFECTION | | 179 | -0.5131591 | -1.8831682 | 0 | 0.00621569 |
| 04620 - TOLL-LIKE RECEPTOR SIGNALING PATHWAY | | 100 | -0.55791724 | -1.8631967 | 0 | 0.00656549 |
| 05142 - CHAGAS DISEASE (AMERICAN TRYPANOSOMIASIS) | | 102 | -0.5557552 | -1.8549004 | 0 | 0.00668411 |
| 04064 - NF-KAPPA B SIGNALING PATHWAY | | 88 | -0.55728894 | -1.8345244 | 0 | 0.00836477 |
| 02010 - ABC TRANSPORTERS | | 44 | -0.6148824 | -1.8323972 | 0.00194175 | 0.00803055 |
| 04668 - TNF SIGNALING PATHWAY | | 110 | -0.5374432 | -1.8296909 | 0 | 0.00778468 |
| 05162 - MEASLES | | 133 | -0.5313655 | -1.8245081 | 0 | 0.00766224 |
| 05164 - INFLUENZA A | | 166 | -0.5018732 | -1.8237127 | 0 | 0.00707283 |
| 05332 - GRAFT-VERSUS-HOST DISEASE | | 36 | -0.6273978 | -1.7797297 | 0 | 0.01181645 |
| 05222 - SMALL CELL LUNG CANCER | | 86 | -0.52572626 | -1.7350847 | 0 | 0.01992926 |
| 03013 - RNA TRANSPORT | | 144 | -0.48264685 | -1.7085238 | 0 | 0.02669033 |
| 05169 - EPSTEIN-BARR VIRUS INFECTION | | 195 | -0.46322137 | -1.7003168 | 0 | 0.0272169 |
| 05160 - HEPATITIS C | | 131 | -0.47697887 | -1.684649 | 0 | 0.03097046 |
| 00970 - AMINOACYL-TRNA BIOSYNTHESIS | | 44 | -0.5643204 | -1.6632261 | 0.00909091 | 0.03819939 |
| 00512 - MUCIN TYPE O-GLYCAN BIOSYNTHESIS | | 30 | -0.5949211 | -1.6480107 | 0.01132076 | 0.04219002 |
| 04920 - ADIPOCYTOKINE SIGNALING PATHWAY | | 69 | -0.5092875 | -1.6330667 | 0.00364299 | 0.04644104 |
| NAME | SIZE | | ES | NES | NOM p-val | FDR q-val |
| RA+THAL - down |  | |  |  |  |  |
| 05322 - SYSTEMIC LUPUS ERYTHEMATOSUS | 115 | | -0.7763048 | -2.6611395 | 0 | 0 |
| 03030 - DNA REPLICATION | 35 | | -0.834228 | -2.3800743 | 0 | 0 |
| 04110 - CELL CYCLE | 123 | | -0.65331 | -2.2379935 | 0 | 0 |
| 05034 - ALCOHOLISM | 166 | | -0.62810785 | -2.229551 | 0 | 0 |
| 05203 - VIRAL CARCINOGENESIS | 197 | | -0.5454169 | -2.0119762 | 0 | 3.07E-04 |
| 03460 - FANCONI ANEMIA PATHWAY | 51 | | -0.650251 | -1.9520069 | 0 | 9.45E-04 |
| 04621 - NOD-LIKE RECEPTOR SIGNALING PATHWAY | 57 | | -0.6037466 | -1.8252383 | 0 | 0.01463543 |
| 05222 - SMALL CELL LUNG CANCER | 86 | | -0.54328173 | -1.7905114 | 0 | 0.02289311 |
| 05166 - HTLV-I INFECTION | 256 | | -0.4712678 | -1.780093 | 0 | 0.02392387 |
| 05020 - PRION DISEASES | 36 | | -0.62444526 | -1.7480744 | 0.00796813 | 0.03186256 |
| 04064 - NF-KAPPA B SIGNALING PATHWAY | 88 | | -0.5229775 | -1.7459716 | 0 | 0.02959343 |
| 05200 - PATHWAYS IN CANCER | 327 | | -0.44553995 | -1.7285562 | 0 | 0.03264257 |
| 05133 - PERTUSSIS | 72 | | -0.5437073 | -1.7242639 | 0.00199203 | 0.03153525 |
| 04114 - OOCYTE MEIOSIS | 110 | | -0.5124084 | -1.7150742 | 0 | 0.03402138 |
| 03440 - HOMOLOGOUS RECOMBINATION | 28 | | -0.64824235 | -1.7125374 | 0.00769231 | 0.03281244 |
| 03430 - MISMATCH REPAIR | 23 | | -0.6628376 | -1.699929 | 0.01006036 | 0.03549273 |
| 04068 - FOXO SIGNALING PATHWAY | 131 | | -0.48733902 | -1.6992987 | 0 | 0.03366178 |
| 05202 - TRANSCRIPTIONAL MISREGULATION IN CANCER | 170 | | -0.4693751 | -1.694626 | 0 | 0.03365058 |
| 05144 - MALARIA | 46 | | -0.57743734 | -1.6878467 | 0.01077586 | 0.03391256 |
| 05161 - HEPATITIS B | 144 | | -0.47521278 | -1.6696259 | 0 | 0.03932544 |
| 05142 - CHAGAS DISEASE (AMERICAN TRYPANOSOMIASIS) | 102 | | -0.50325304 | -1.6666713 | 0 | 0.03891586 |
| 05134 - LEGIONELLOSIS | 54 | | -0.56260717 | -1.666066 | 0.00204082 | 0.03733853 |
| 05332 - GRAFT-VERSUS-HOST DISEASE | 36 | | -0.5894024 | -1.6575265 | 0.01422764 | 0.03890139 |
| 05219 - BLADDER CANCER | 38 | | -0.5815643 | -1.6575208 | 0.00408163 | 0.0372805 |
| 04620 - TOLL-LIKE RECEPTOR SIGNALING PATHWAY | 100 | | -0.49181256 | -1.6472642 | 0.002079 | 0.0403164 |
| 05223 - NON-SMALL CELL LUNG CANCER | 56 | | -0.53050184 | -1.6463935 | 0.00793651 | 0.03904773 |
| 04668 - TNF SIGNALING PATHWAY | 110 | | -0.48281643 | -1.6358861 | 0 | 0.04165724 |
| 00240 - PYRIMIDINE METABOLISM | 99 | | -0.4883406 | -1.6301312 | 0.00393701 | 0.0424618 |
| 03410 - BASE EXCISION REPAIR | 33 | | -0.57902664 | -1.6280426 | 0.01649485 | 0.04167321 |

Supplementary Table S1. Gene Set Enrichment analysis of pathways differentially regulated in U251 xenografts treated with RA and THAL as sole agents, or in combination, RA+THAL, relative to untreated controls. SIZE: number of genes in the pathway; ES: enrichment score; NES: normalized enrichment score; NOM p-val: nominal p-value; FDR q-val: false discovery rate q-value;
